# Disability associated with mental disorders

**DOI:** 10.4103/0019-5545.31597

**Published:** 2006

**Authors:** Pranit K. Chaudhury, Kamala Deka, Dhrubajyoti Chetia

**Affiliations:** * Professor and Head Department of Psychiatry, Assam Medical College and Hospital, Dibrugarh; ** Associate Professor Department of Psychiatry, Assam Medical College and Hospital, Dibrugarh; *** Postgraduate Teacher Department of Psychiatry, Assam Medical College and Hospital, Dibrugarh

**Keywords:** Mental disorders, disability, assessment, IDEAS

## Abstract

**Background:**

Disability associated with mental illness is a major contributor to the global burden of disease. The present study looks at some aspects of disability associated with 7 psychiatric disorders: schizophrenia, bipolar affective disorder, anxiety disorders, depression, obsessive–compulsive disorder, dementia, and mental and behavioural disorders due to the use of alcohol.

**Aims:**

(i) To evaluate the nature and quantity of disabilities in the study groups; (ii) to compare the degree of disability with the severity of the disorder; (iii) to compare disability among various disorders; and (iv) to study the longitudinal stability of disability in the disease groups.

**Methods:**

A total of 228 patients attending the OPD, Department of Psychiatry, Assam Medical College, Dibrugarh, between July 2003 and June 2004, who were diagnosed as per ICD-10 guidelines and SCAN, were included in the study. Severity was assessed by the application of some commonly used rating scales for each specific disorder. The level of disability was assessed by using the Indian Disability Evaluation and Assessment Scale (IDEAS). Patients were followed up at 6 and 12 months. Statistical analysis was done on SPSS version 10.

**Results:**

All the 7 disorders under study are associated with significant disability; schizophrenia being maximally disabling. Disability associated with alcohol use disorder and anxiety is comparable to disability on account of OCD. Over a period of 12 months, disability due to depression, alcohol use disorder and anxiety tend to remain significant.

## INTRODUCTION

Defining disability is not an easy task, and it is becoming clear that no single definition can cover all aspects of disabilities. According to the International Classification of Impairment, Disability and Handicap (ICIDH, 1980), disability is interference with activities of the whole person in relation to the immediate environment.^1^

Though critics skeptical of the disabling tendencies of psychiatric disorders abound, it is a well-accepted fact that the mentally ill constitute a sizeable chunk of the disabled population. Within the ambit of the definition of disability under the Persons with Disabilities Act, 1995, mental illness means a ‘disorder of the mind that results in partial or complete disturbance in the person's thinking, feeling and behaviour which may also result in recurrent or persistent inability or reduced ability to carryout activities of daily living, self-care, education, employment and participation in social life’.^2^ These disorders include schizophrenia, obsessive–compulsive disorder (OCD), bipolar disorder and moderate-to-severe depression of at least 3 years' duration with proof of continuous treatment.^3^

Disability associated with mental illness is a major contributor to the global burden of disease. As per the National Sample Survey Organization (NSSO) 1991 statistics,^4^ 1.9% of India's population is disabled in one way or the other. Study of disability associated with mental disorders therefore becomes a matter of prime importance.

The present study aimed (i) to evaluate the *nature* and *quantity* of disabilities in the study groups; (ii) to compare the *degree* of disability with the *severity* of the disorder; (iii) to compare disability among the various disorders; and (iv) to study the *longitudinal stability* of disability in the disease groups.

## METHODS

Recruitment to the study started in July 2003 in the outpatient wing of the Department of Psychiatry, Assam Medical College, Dibrugarh and continued till June 2004.

*Inclusion criteria:* (i) age range: 18 years and above; (ii) sex: both sexes were included; and (iii) patients diagnosed of the disorders under study as per ICD-10 criteria.

*Exclusion criteria:* (i) persons with mental retardation; (ii) chronic debilitating illness; (iii) organic brain disease *(other than dementia and alcohol use disorder);* and (iv) psychiatric co-morbidity.

### Procedure

Every second patient presenting to the outpatient wing was assessed and diagnosed as per ICD-10 criteria. Once a clinical diagnosis was made and, if the inclusion criteria were fulfilled, the patient was counselled to be a part of the study and his/her consent obtained. The patient was then reassessed with the SCAN to validate the clinical diagnosis and eliminate co-morbid psychiatric conditions. Of the total study group of 228 patients, 156 (68.4%) were males and 72 (31.6%) females.

Seven disorders, namely, schizophrenia, bipolar affective disorder (BAD), depressive episodes (MDD), anxiety disorder, OCD, dementia and mental and behavioural disorder due to use of alcohol (AUD), as per ICD-10 criteria were studied. The relevant subscale was then applied to assess the severity of the disorder. Disability on account of the illness was assessed through the disability scales (IDEAS) and the scores so obtained were compared with the disorder severity to establish causal relationship between the two.

### Diagnostic tools

- *ICD-10:* The clinical OPD diagnosis was based on the diagnostic criteria laid down in the *ICD-10 classification of mental and behavioural disorders—Clinical description and diagnostic guidelines.*^5^
- *SCAN:* The Structured Clinical Assessment in Neuropsychiatry^6^ (SCAN) was used to validate the clinical diagnosis and eliminate co-morbid psychiatric conditions.

### Assessment of severity

The assessment of severity of specific disorders was done by application of commonly used rating scales for each specific disorder. The tools used were: Hamilton's Rating Scale for Anxiety,^7^ Hamilton's Rating Scale for Depression,^8^ AUDIT questionnaire for alcohol-related disorders,^9^ Positive and Negative Symptoms Scale (PANSS)^10^ for schizophrenia, Mini Mental State Examination (MMSE)^11^ for dementia, the Young Mania Rating Scale^12^ and the Hamilton Rating Scale for Depression^13^ for bipolar affective disorders, and The Yale Brown Obsessive Scale^14^ (Y-BOCS) for OCD.

### Evaluation of disability

The following two scales were used to assess the level of functionality in order to determine the amount of disability.

- *IDEAS:* Each subject in the study groups was assessed for disability due to the current illness using the Indian Disability Evaluation and Assessment Scale^15^ (IDEAS) developed by the Rehabilitation Committee of the Indian Psychiatric Society. IDEAS quantify disability by relying on information in four core areas of self-care, interpersonal relationships, communication and understanding and work functioning. The cut-off point separating mild to moderate disability from severe disability according to this scale is a Global Disability score of 8 corresponding to ≥40% disability.
- *The London Handicap scale:* The London Handicap scale^16^ is a widely used tool that measures the level of functional impairments in an individual taking into account the discomforts on account of both physical and social factors.

### Follow-up

Disability on account of the disorder was measured at three points of time—at recruitment, and after 6 and 12 months.

### Data analysis

Statistical analysis was done using the Statistical Package for Social Sciences, version 10 (SPSS-10).

## RESULTS

Table 1 shows that there were 70 patients (31%) with a diagnosis of schizophrenia; 30 patients (13%) each with a diagnosis of bipolar affective disorder (BAD), anxiety disorders, depression (MDD) and alcohol use disorder (AUD); 25 patients (11%) with OCD and 13 (6%) patients with dementia. A majority of patients in all diagnostic groups except dementia hailed from rural areas (Table 2). The diagnostic groups were fairly comparable in terms of mean age, duration of illness and the mean years of formal education received (Table 3).

**Table 1:** Distribution of the diagnostic groups in accordance with sex

Disorder	Mile		Female		Total	
			
	*n*	%	*n*	%	*n*	%
Schizophrenia	46	65.7	24	34.3	70	31
Depression (MDD)	19	63.3	11	36.7	30	13
BAD	22	73.3	8	26.7	30	13
Anxiety disorders	20	66.7	10	33.3	30	13
AUD	30	100	Nil	0	30	13
OCD	10	40	15	60	25	11
Dementia	9	69.2	4	31.8	13	6
Total	156	68.4	72	31.6	228	100

BAD - Bipolar affective disorder; AUD - Alcohol use disorder; OCD - Obsessive-compulsive disorder

**Table 2:** Distiibution of the diagnostic groins in accoidance with domicile

Disorder	Uban		Rural	
		
	*n*	%	*n*	%
Schizophrenia	14	20	56	80
Depression (MDD)	11	36.7	19	63.3
BAD	13	43.3	17	56.7
Anxiety disorders	5	16.7	25	83.3
AUD	9	30	21	70
OCD	12	48	13	52
Dementia	10	76.9	3	23.1
Total	74	32.5	154	67.5

BAD - Bipolar affective disorder; AUD - Alcohol use disorder; OCD - Obsessive-compulsive disorder

**Table 3:** Age, education and duration of illness (mean ± SD in years)

Disorder	Mean age	Mean education	Mean duration
Schizophrenia	31.43 ± 9.42	8.40 ± 5.237	7.07 ± 5.39
Depression (MDD)	39.87 ± 11.03	4.66 ± 4.43	10.75 ± 3.94
BAD	38.97 ± 14.98	9.10 ± 4.60	13.13 ± 9.58
Anxiety disorders	35.5 ± 10.54	6.10 ± 5.45	10.10 ± 4.81
AUD	41.23 ± 8.32	9.43 ± 3.6	14 ± 7.79
OCD	32.88 ± 10.29	9.76 ± 3.27	5.88 ± 4.46
Dementia	65.92 ± 8.65	8.77 ± 3.99	2.08 ± 1.38

BAD - Bipolar affective disorder; AUD - Alcohol use disorder; OCD - Obsessive-compulsive disorder

Sixty-four per cent of patients suffering from schizophrenia had severe disability as per the IDEAS-GS. This fraction in depression and bipolar affective disorders were 33.3% and 30%, respectively. Alcohol-related disorders and anxiety disorder patients had equal proportion of patients with severe disability (16.7% each). Similarly, 16% and 69.2% of patients suffering from OCD and dementia, respectively, were severely disabled (Table 4).

**Table 4:** Distribution of patients with severe disability in individual disease groups

Disorder	Total	IDEAS-GS≥	%
Schizophrenia	70	45	64.3
Depression (MDD)	30	10	33.3
BAD	30	9	30.0
Anxiety disorders	30	5	16.7
AUD	30	5	16.7
OCD	25	4	16.0
Dementia	13	10	69.23
Total	228	88	38.59

BAD - Bipolar affective disorder; AUD - Alcohol use disorder; OCD - Obsessive-compulsive disorder

### Disability in different diagnostic groups

#### Schizophrenia

The schizophrenia group consisted of 70 patients, of whom 80% were from a rural background. The mean age of this sample was 31.43±9.42 years, the mean duration of illness 7.07±5.39 years and the mean years of formal education completed in the group was 8.40±5.237 (Table 3).

Correlation studies to measure the relation of disability on the IDEAS scale to the severity of illness as measured by PANSS showed a strong positive correlation with IDEAS-GS on all the three components of the PANSS scale. This correlation was seen to be stronger for the Negative syndrome scores (r=0.610, p<0.01), then for the Positive syndrome scores (r=0.322, p<0.01). The correlation between the General Psychopathology scores and IDEAS Global score was also highly significant (r=0.518, p<0.01) (Table 5).

**Table 5:** Correlation between rating scales and disability

	IDEAS-GS		LHS	
		
Disorder and rating scales	Correlation coefficient	Significance (2-tailed)	Correlation coefficient	Significance (2-tailed)
*Schizophrenia*				
PANSS-NS	0.610**	0.000	0.606**	0.000
PANSS-PS	0.322**	0.007	0.329**	0.005
PANSS-GPS	0.518**	0.000	0.627**	0.000
*Depression (MDD)*				
HAM-D	0.531**	0.003	0.660**	0.000
*Anxiety*				
HAM-A	0.530**	0.002	0.560**	0.001
*BAD*				
YMRS	0.633**	0.000	0.599**	0.001
*AUD*				
AUDIT TS	0.357*	0.026	0.060	0.30
*OCD*				
Y-BOCS-CS	0.411*	0.41	0.555**	0.004
Y-BOCS-CS	-0.030	0.885	-0.066	0.753
Y-BOCS-CS	0.071	0.737	0.141	0.502
*Dementia*				
MMSE	-0.550*	0.026	-0.755**	0.001

* Correlation is significant at the 0.05 level

** Correlation is significant at the 0.01 level

[IDEAS-GS - Indian Disability Evaluation and Assessment Scale, Global Score; PANSS - Positive and Negative symptoms scale; NS - Negative syndrome; PS- Positive syndrome; GPS - General psychopathology; HAM-D - Hamilton's Rating Scale for Depression; HAM-A - Hamilton's Rating Scale for Anxiety; AUDIT questionnaire for Alcohol-related disorders, MMSE - Mini Mental State Examination; YMRS - Young Mania Rating Scale; Y-BOCS - Yale Brown Obsessive Scale; OS - Obsession score; CS - Compulsion score; TS - Total score; BAD - Bipolar affective disorder; AUD - Alcohol use disorder; OCD - Obsessive- compulsive disorder

Similar results were obtained on the London Handicap Scale (Table 5). On the London Handicap Scale, correlation studies returned correlation coefficients of 0.606, 0.329, and 0.627 with NS, PS, and GPS, respectively, all correlations significant at p<0.01. Forty-five patients (64.3%) had disability more than 40% on the IDEAS scale. On the individual components of the IDEAS scale, correlation tests with the components of the PANSS scale showed that while both GP and NS seem to affect all areas, PS do not significantly affect communication and self-care activities (Table 6).

**Table 6:** Areas of functioning impaired by different disorders- Correlation between rating scale scores and areas of functioning (correlation coefficients with significance are shown in parenthesis)

Disorder	Rating scale	IDEAS			
		
		Self-care	IPR	Comm	Work
Schizophrenia	PANSS-NS	0.474**	0.433**	0.373**	0.555**
		(0.000)	(0.000)	(0.001)	(0.000)
	PANSS-PS	0.152	0.366**	0.161	0.350**
		(0.211)	(0.002)	(0.182)	(0.003)
	PANSS-GPS	0.316**	0.428**	0.273*	0.500**
		(0.008)	(0.000)	(0.022)	(0.000)
Depression (MDD)	HDRS	0.492**	0.325	0.315	0.590**
		(0.006)	(0.080)	(0.090)	(0.001)
Anxiety disorders	HAM-A	0.280	0.387*	0.383*	0.458*
		(0.134)	(0.034)	(0.036)	(0.011)
BAD	YMRS	0.411*	0.610**	0.587**	0.510**
		(0.027)	(0.000)	(0.001)	(0.005)
AUD	AUDIT-TS	0.045	0.253*	0.190	0.165
		(0.383)	(0.049)	(0.105)	(0.124)
OCD	Y-BOCS-OS	0.271	0.341	0.360	0.431*
		(0.190)	(0.095)	(0.077)	(0.031)
	Y-BOCS-CS	-0.090	0.193	0.000	-0.138
		(0.669)	(0.354)	(1.000)	(510)
Dementia	MMSE	-0.356	-0.590*	-0.531*	-0.421
		(0.116)	(0.017)	(0.031)	(0.076)

* Correlation is significant at the 0.05 level

** Correlation is significant at the 0.01 level

[IDEAS-GS - Indian Disability Evaluation and Assessment Scale; PANSS - Positive and Negative symptoms scale; NS - Negative syndrome; PS- Positive syndrome; HAM-D - Hamilton's Rating Scale for Depression; HAM-A - Hamilton's Rating Scale for Anxiety; AUDIT questionnaire for Alcohol-related disorders, MMSE - Mini Mental State Examination; YMRS - Young Mania Rating Scale; Y-BOCS - Yale Brown Obsessive Scale; Y-BOCS-OS - Obsession score; Y-BOCS-CS - Compulsion score; TS - Total score; IPR- Interpersonal relationships COMM- Communication; BAD - Bipolar affective disorder; AUD - Alcohol use disorder; OCD - Obsessive-compulsive disorder

#### Depression

The depressive disorder group consisted of 30 patients primarily from rural background with group mean age of 39.87±11.03 years, the mean duration of illness 4.66±4.43 years and the mean years of formal education completed at 10.75±3.94 years (Table 3). Correlation analysis between HAM-D and IDEAS Global Score showed a highly significant positive correlation (r=0.531, p<0.01). An even stronger positive correlation was noted between IDEAS Global Score and the London Handicap Scale (r=0.660. p<0.01) (Table 5).

On individual areas of functioning, it was observed that severity of depression significantly affected self-care and work, while interpersonal relations and communication were less affected by depression. No significant correlation being found between HDRS scores and these two areas of functioning (Table 6).

#### Anxiety disorder

The anxiety disorder group comprised of 30 patients suffering from GAD and Panic disorders. The mean age of this sample was 35.5±10.54 years, the mean duration of illness 6.10±5.45 years and the mean years of formal education completed in the group was 10.10±4.81 years (Table 3). Correlation tests showed a positive correlation between the severity of anxiety as measured on the Hamilton Anxiety rating scale and both the IDEAS and the LHS (Table 5). This correlation, it was observed was more significant in the LHS (r=0.560, p<0.05) then in IDEAS (r=0.530, p<0.01).

One observation in the analysis of the effect of severity on the core areas of functioning in the IDEAS scale was that while deterioration in the level of functioning in all the areas appeared to have a positive correlation to the severity of illness, maximal deterioration was in the area of work (r=0.458, p<0.05). On the other hand self-care, though positively correlated was not significantly affected by the severity of anxiety symptoms (Table 6).

#### Bipolar disorders

Thirty patients with a diagnosis of bipolar affective disorder were included in this group. The mean age of this sample was 38.97±14.98 years, the mean duration of illness 13.13±9.58 years and the mean years of formal education completed in the group was 9.10±4.60 years (Table 3). Correlation of severity of bipolar affective disorders and the disability measurement tools revealed a positive correlation on the IDEAS global score as well as the LHS total score (Table 5). The correlation tests returned values of 0.633 and 0.599, respectively on the two scales.

All the core areas of functioning, i.e. self-care, interpersonal relations, communication and work were affected in bipolar disorders. Within these independent facets of functioning, self-care, though significant was the area that was least affected (Table 6).

#### Alcohol use disorder

This group comprises of thirty patients, 70% of them from a rural background, all males, with a mean age of 41.23±8.32 years; mean duration of illness of 14±7.79 and mean years of formal education completed was 9.43±3.6 years (Table 3). A positive correlation between AUDIT score and IDEAS-GS and AUDIT score indicates the tendency of alcohol of causing impairment of functioning. (Table 5) 16.7% of the sample had disability more than 40%. However, the same was not reflected in the London Handicap Scale.

The main area of functioning significantly influenced by alcohol included is interpersonal relations (r=0.253, p<0.05), (Table 4). The other components also bear a weak positive correlation but no statistically significant correlation was found. Self-care appears to be the area, which is least, affected. Similar results were obtained in correlation with LHS scores as well.

#### Obsessive-compulsive disorder

The OCD patients numbered twenty-five, with 60% of them being females and 52% of the sample hailing from an urban background. The mean age of this sample was 32.88±10.29 years, the mean duration of illness 5.88±4.46 years and the mean years of formal education completed in the group was 9.76±3.27 years (Table 3). The severity of illness was measured with Y-BOCS and three scores were obtained that measured severity of Obsession and Compulsions separately. The sum of Compulsion Score (CS) and Obsession Score (OS) is labeled Total Score (TS). Correlation studies were done with the disability measuring instruments separately for all these three scores (Table 5).

Correlation study results indicate that the severity of obsessions (Y-BOCS-OS) have a significant positive correlation on disability. However compulsions (indicated by Y-BOCS-CS) tend to have a weak negative correlation on disability scores. The correlation between disability and total Y-BOCS score, though positive was not statistically significant. It was observed that work was the area of functioning to be significantly impaired due to obsessions. The other areas though affected did not appear to be significantly impaired. With compulsion a negative correlation, though not significant was observed in the areas of work and self-care (Table 6).

#### Dementia

The mean age of the thirteen patients that comprised the dementia group was 65±8.65 years, the mean duration of illness 2.08±10.4 years and the mean years of formal education was 8.77±3.92 years. Most of the patients (76.9%) belonged to an urban background, and this sample was predominantly male (69.2%) (Table 3). The relation of MMSE scores to cognitive deterioration is a negative one. The same is reflected in the correlation tests between MMSE scores and IDEAS-GS as well as between MMSE scores and LHSTS. The correlation appeared stronger on the London Handicap scale (r=–0.755, p<0.001) then with The IDEAS (r=–0.550, p<0.05) (Table 5).

Correlation of the individual components of IDEAS to the severity of illness shows that interpersonal relationship and communication appeared to be the more important contributors to disability in comparison to work and self-care. (Table 6)

### Disability across groups

Comparison of disability in the different disorders was done applying ANOVA on SPSS 10. The results of this test are given in Tables 7 and 8. The results indicate that schizophrenia is by far the most disabling of the mental disorders, followed by dementia. Depression, OCD, Bipolar affective disorders and Alcohol use disorders are the next four disability causing disorders in order of their severity of disability caused. Anxiety disorders cause the least amount of disability.

**Table 7:** ANOVA table

	Sum of squares	DF	Mean square	F	P
Between groups	1475.721	6	245.954		
Within groups	2844.011	221	12.869}	19.112	0.000
Total	4319.732	227		

**Table 8:** Multiple comparisons (post-hoc test); dependent variable: IDEAS global score

Diagnosis (I)	Diagnosis (J)	Mean difference (I-J)	Standard error	P
Depression	Schizophrenia	-3.029 (*)	0.796	0.001
Anxiety disorder	Schizophrenia	-6.201 (*)	0.770	0.000
OCD	Schizophrenia	-4.809 (*)	0.850	0.000
BAD	Schizophrenia	-5.187 (*)	0.787	0.000
Dementia	Schizophrenia	-1.529	1.233	0.733
AUD	Schizophrenia	-5.429 (*)	0.796	0.000

* The mean difference is significant at the 0.05 level. BAD - Bipolar affective disorder; AUD - Alcohol use disorder; OCD - Obsessive-compulsive disorder

### Stability of disability

Follow-up of the patients in the study to measure the stability of disability over time was done through scheduled appointments for assessment in terms of status since recruitment at two points, six months apart. The percentage of the study sample attending the 6-month follow up was 54.82% while 20.61% attended the OPD for the second follow up 12 months after recruitment.

**Figure 1:**
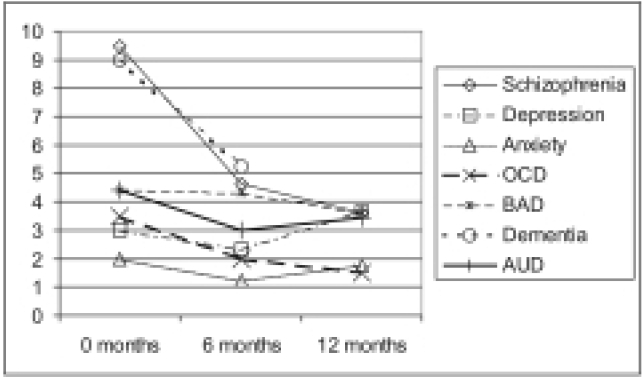
Change in mean IDEAS score at 6 and 12 months. IDEAS-GS BAD - Bipolar affective disorder; AUD - Alcohol use disorder; OCD - Obsessive-compulsive disorder

Table 9 shows the number of patients who came for follow up at the end of 6 months and at 12 months in the different diagnostic groups. The change in the IDEAS score in patients attending the second follow up is depicted in Fig. 1.

**Table 9:** Follow-up at 6 and 12 months

Disoider	0 months	6 months		12 months	
			
		*n*	%	*n*	%
Schizophrenia	70	43	61.42	19	27.14
Depression	30	8	26.6	3	10.00
BAD	30	23	76.6	10	33.33
Anxiety disorders	30	21	70.0	4	13.33
AUD	30	16	53.33	7	23.33
OCD	25	10	40.00	4	16.00
Dementia	13	4	30.7	Nil	-
Total	228	125	54.82	47	20.61

BAD - Bipolar affective disorder; AUD - Alcohol use disorder; OCD - Obsessive-compulsive disorder

The interpretation of the graphs has to be done keeping in mind the small number of patients and also the fact that for chronic conditions such as mental disorders, a one-year period is too short a time to give a definite conclusion on the outcome of the disorder. The figure therefore should only be seen as an indication of the trend of progress or otherwise of impairments in functioning. The disability associated with all the diseases show a reduction in severity at the end of 6 months. While impairments associated with schizophrenia, OCD and BAD tend to even out after 6 months, the impairments in the other 3 conditions, i.e. anxiety disorder, Depression and AUD appear to increase slightly indicating that impairments associated with these disorders is not negligible at least over a period of 12 months. There was no significant difference in disability among the groups at the end of 12 months.

## DISCUSSION

Our study sample was predominantly from the rural areas surrounding Dibrugarh district of Assam, was predominantly male except in the OCD group where there was a female predominance and the domiciliary status was nearly equal in both the sexes. The mean age of the patients in the different diagnostic categories was in the fourth decade except understandably for Dementia where the mean age was 65.92 years. The educational background was comparable in all the disease categories except in depression where the mean years of formal education was 4.66 years. The minimal duration of illness was at least 5 years except in dementia. Dementia patients appeared to present within 2 years of onset of illness. However this group comprised only 6 % of the entire study group.

Our findings indicate that significant amount of disability occur in a varying proportion of patients in all the disorders under study. The degree of disability tends to correlate with the severity of the disorder as reflected in the rating scales, although not all associations were significant when measured in terms of areas of functioning impaired on account of the disorder. This is a replication of studies done earlier.^17^

On a proportionate basis, dementia and schizophrenia top the list of disability causing disorders. The difference between these two groups in our study was that while schizophrenia tends to significantly affect all the four core areas of functioning, dementia was found to have a detrimental bearing primarily in the areas of interpersonal relations and communi-cation. This however does not mean that self-care and work functioning remain unaffected by dementia. The results need to be interpreted keeping in mind that the sample size in the two diagnostic groups was not equal and that the dementia patients presented to the clinic relatively earlier than the schizophrenia patients did. This finding is in contrast to that of Sanderson and Andrews,^17^ who in their study on prevalence and severity of mental health-related disability, found depression and anxiety disorders to be the two primary disorders associated with disability.

In our study, the percentage of individuals with depression and bipolar disorders with severe disability were similar at 33.33% and 30%, respectively. The primary area of dysfunction in depression was in the areas of self-care and work. Bipolar disorder, such as schizophrenia, tends to cause dysfunction in all four core areas of functioning.

Both bipolar disorders and depression have been implicated in causing disability in several studies. One recent example is the study done in the urban population by Olfson *et al.,*^18^ who found depression and bipolar disorder to be the two major disability-related disorders.

More than 40% disability on the IDEAS scale was seen in 16.7%, 16.0% and 16.7% of patients with Alcohol use disorders, OCD and anxiety disorders, respectively. The primary areas of dysfunction in the three groups differed. OCD was associated with work dysfunction; alcohol affects mainly interpersonal relations, while anxiety affects three areas viz. interpersonal relations, communication and work. Earlier studies^19^,^20^ were similarly able to show the disabling propensity of OCD. Unlike Olfson *et al.'s*^18^ study which failed to correlate GAD with disability, Kessler *et al.*^21^ had demonstrated that panic disorder is independently associated with greater dysfunction. The difference in the degree of dysfunction was done by analysis of variance of mean with IDEAS-GS as the dependent variable. The results indicate that schizophrenia is by far the most disabling of the mental disorders, followed by dementia.

Depression, OCD, BAD and AUD are the next four disability-causing disorders in order of their severity of disability caused. Anxiety disorders cause the least amount of disability, which is different from the finding of Bassett *et al.,*^22^ who found affective and anxiety disorders to be more disabling than substance use disorder.

It becomes difficult to comment on disability when a small number of patients came for follow-up. Assessed from those few patients who did turn up, it does indicate a tendency for dysfunction to remain static beyond 6 months of treatment. With AUD, anxiety disorder as well as depression, there is an observed tendency for IDEAS-GS to marginally increase after 6 months in spite of treatment. Only further follow-up can provide data on the stability of dysfunction associated with the disorders under study. However, the present study being of limited size and entirely hospital-based is not representative of the community at large

## LIMITATIONS

The sample size in the present study was not equally distributed across the various diagnostic groups. Some aspects of the disorders, such as duration of illness, socioeconomic status, pre-morbid personality and sex have not been correlated with the disability. Far too few patients attended the second follow-up, which made it difficult to comment on the stability of disability.

## CONCLUSION

All 7 disorders under study are associated with significant disability; schizophrenia being maximally disabling. On the IDEAS scale, it was noted that the areas of functioning impaired on account of the disorder varied by diagnosis. It was also seen that disability associated with AUD and anxiety is comparable to disability on account of OCD. Short-term follow-up over 12 months showed that disability due to depression, AUD and anxiety tends to remain significant.

Considering the fact that psychosocial management is an important component of psychiatric care, knowledge of specific areas of dysfunction may have implications for treatment. If alleviation of disability is a goal of treatment, greater focus need to be given to specific areas of functioning in different disorders. The finding that the severity of disability varies by diagnosis also suggests that IDEAS is a sensitive tool in detecting difference in disability, both qualitatively and quantitatively across different types of mental disorder.
